# Investigating Tumor-Infiltrating Lymphocytes in the Microenvironment of Oral Squamous Cell Carcinoma (OSCC) and Oral Potentially Malignant Disorders (OPMDs): Can They Shift Our Perspective? A Scoping Review

**DOI:** 10.3390/jcm14020606

**Published:** 2025-01-18

**Authors:** Samuele Sutera, Olga Anna Furchì, Monica Pentenero

**Affiliations:** Oral Medicine and Oral Oncology Unit, Department of Oncology, University of Turin, 10043 Turin, Italy; olgafurchi@gmail.com (O.A.F.); monica.pentenero@unito.it (M.P.)

**Keywords:** tumor microenvironment, tumor-infiltrating lymphocytes, oral squamous cell carcinoma, oral potentially malignant disorder, malignant transformation, carcinogenesis, prognostic biomarker

## Abstract

**Background/Objectives**: Tumor-infiltrating lymphocytes (TILs) play a crucial role in the tumor microenvironment (TME), influencing the progression, prognosis, and response to treatment in oral squamous cell carcinoma (OSCC) and its precursors, oral potentially malignant disorders (OPMDs). This scoping review assesses the current literature on TILs in the TME of OSCC and OPMDs, aiming to identify trends and gaps in the research. **Methods**: A comprehensive search was performed in PubMed, using the following query terms: “Tumor Microenvironment AND (mouth neoplasms OR oral lichen OR leukoplakia OR oral lichenoid OR dysplasia OR GVHD OR lupus)”. Based on the inclusion criteria, we selected in vivo human original research and clinical observational studies that focused on TILs within the TME of OSCC and OPMDs. **Results**: Out of 1152 results in PubMed, 58 studies were selected and analyzed. These studies investigated various TILs, including T cells, B cells, and natural killer (NK) cells. Of these, 47 studies focused on the OSCC TME, 4 examined the OPMDs ME, and 7 compared OSCC TME and OPMDs ME. **Discussion**: While TILs in OSCC have been extensively studied, research on infiltrating lymphocytes in OPMDs is still limited. In OSCC, CD8+ T cells, T helper 1 cells, and NK cells are associated with strong antitumor activity, whereas CD4+ T cells, including T helper 2 and regulatory T cells, are linked to protumoral effects. B cells remain less explored due to their low frequency in the TME. In OPMDs, trends suggest an increase in activated CD8+ T cells in OLP and lower NK cell numbers compared to OSCC, which may contribute to malignant transformation. Understanding the spatial distribution and activation status of TILs within the TME is essential for deciphering their role. The variability in TIL composition highlights the complexity of the TME. **Conclusions**: Current knowledge remains preliminary, though it highlights the crucial role of TILs in carcinogenesis and OSCC. A more in-depth understanding could improve diagnostic and therapeutic strategies, including the assessment of the risk of malignant transformation in OPMDs.

## 1. Introduction

Since the 1970s, the role of immune cells infiltrating the tumor microenvironment (TME) in tumor development and progression has been debated. Although the interaction between tumors and the host immune system is widely recognized, to date, the underlying mechanisms are not fully understand yet.

The relationship between cancer and inflammatory infiltrates has been investigated in several tumor types, including oral squamous cell carcinoma (OSCC), which will be discussed specifically in this paper.

When discussing oral carcinogenesis, oral potentially malignant disorders (OPMDs) must be considered. OPMDs are globally recognized as one of the most importat risk factors for OSCC, alongside tobacco and alcohol consumption. Among OPMDs, the most common include oral leukoplakia (OL), oral lichen planus (OLP), graft-versus-host disease (GVHD), proliferative verrucous leukoplakia (PVL), systemic lupus erythematosus (SLE), discoid lupus erythematosus (DLE), and actinic cheilitis (AC). Oral epithelial dysplasia (OED) does not represent a separate entity but should be considered a step in the carcinogenesis process [1].

To date, the role of the OPMDs microenvironment (ME) in managing OPMDs has not been established. Management still relies on lesion-related features, patient-related factors, and histological examination of the epithelial compartment [2,3].

The emerging evidence highlights how the ME shows a positive gradient in inflammatory infiltrate from non-neoplastic lesions to OED to OSCCs’ TME. Furthermore, how ME changes in patients with a prior history of OSCC and dysplasia appears to be linked with an increased risk of new cancer development [4,5]. Furthermore, ME characterization is fundamental; based on its composition, ME could exert both pro or anti tumoral activity. Generally speaking, tumors characterized by a lack of proinflammatory cells and antitumor activity are referred to as “cold” tumors. In contrast, “hot” tumors have a TME rich in cells and molecules that can attack and kill tumor cells, indicating a strong immune response [6]. Nevertheless, further research is needed to better understand the role of TME.

In the TME, one of the main cell types is represented by the lymphocyte, on which the present review is focused. In this context, they are called tumor-infiltrating lymphocytes (TILs), which include intratumoral TILs and stromal TILs with different prognostic roles. Their importance has been widely observed not only in OSCC but also in several neoplasms, including breast, colon, lymphoma, and gastric cancers. The number, subpopulations, and locations of TILs have been associated with tumor behavior, response to therapy, and prognosis [7].

In addition to qualitative and quantitative assessments of the inflammatory infiltrate, factors such as molecular crosstalk between cancer cells and TME components, as well as escape mechanisms (the tumor’s ability to evade immunological attack), are crucial for prognosis and tumor progression [8,9,10].

Of note, it has been observed that immune system overall is significantly altered in a negative way in patients with carcinoma, and not just in the cancerous tissues. Indeed, tumor-free tissues from patients with cancer show an altered inflammatory infiltrate [11]. Moreover, impaired lymphocytes have been detected in circulating peripheral blood mononuclear cells as well as in TILs, and these impairments are correlated with the biological and pathological characteristics of the tumor [12,13,14]. These evidences should be considered when developing new therapeutic strategies aimed at providing comprehensive care for oncologic patients.

Based on this background, we believe that greater understanding of the oral cancer immune microenvironment could lead to better patient evaluation, improved prognosis, and the development of new therapeutic strategies, such as immunotherapies. Therefore, we conducted a scoping review to assess the current knowledge focusing on the role of TILs in the TME of OSCC and OPMDs, examining their influence from carcinogenesis to tumor dissemination.

## 2. Materials and Methods

A scoping review was conducted following the guidelines of the PRISMA-ScR Checklist [15].

A comprehensive search was conducted in PubMed using the query terms: “Tumor Microenvironment AND (mouth neoplasms OR oral lichen OR leukoplakia OR oral lichenoid OR dysplasia OR GVHD OR lupus).” The search was limited to studies published up to and including May 2024.

The aim of this scoping review was to broadly explore the available literature regarding the role of TILs within the TME of OSCC and OPMDs and map the current evidence in the field. Therefore, the search was limited to the PubMed database as it provides a wide-ranging and comprehensive collection of biomedical studies relevant to our research focus, including articles indexed in MEDLINE.submucosal fibrosis.

We included original human in vivo studies that examined TILs in the context of OSCC and OPMDs. Only full-text articles published in English were considered for inclusion. The exclusion criteria comprised reviews, studies that focused exclusively on the general microenvironment without a specific analysis of TILs, studies unrelated to OSCC or OPMDs, or those where data from the oral cavity could not be distinguished from other anatomical regions (e.g., the pharynx). Additionally, in vitro studies and research based on animal models were excluded (Table 1).

Two review authors (S.S. and M.P.) independently screened the titles and abstracts for relevance. Full texts of potentially relevant studies were then reviewed to determine eligibility. Any disagreements were resolved by discussion.

In each paper, we collected data on TIL markers, mediators of intercellular crosstalk, clinical–pathological endpoints related to OSCC, and the role of TILs in OPMDs (when available). The data were then synthesized to provide a comprehensive overview of the role of TILs in OSCC and OPMDs.

The quality of the included studies was assessed using criteria based on study design, sample size, methods, and reporting. However, due to the heterogeneity of the studies, a formal meta-analysis was not feasible.

## 3. Results

**Figure 1:**
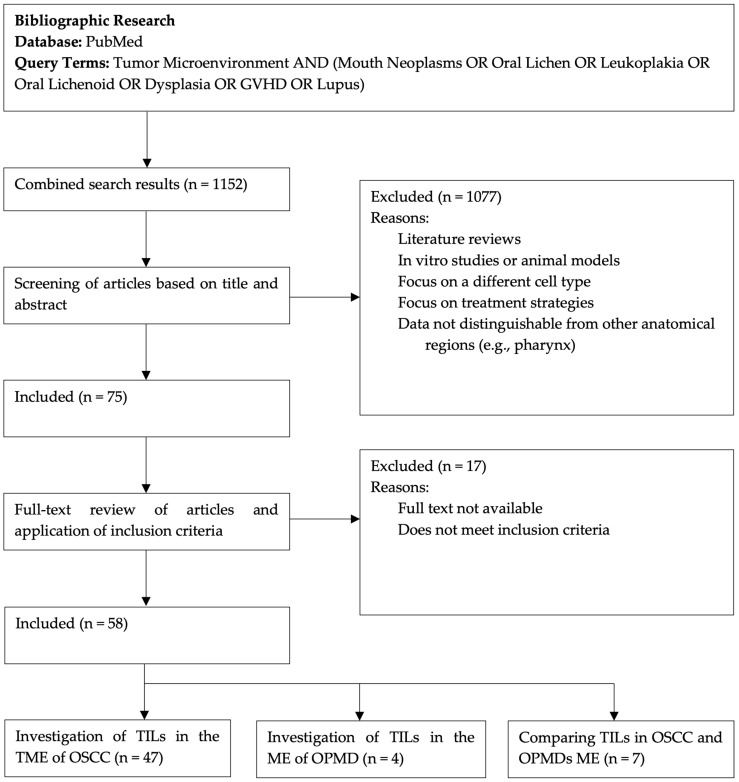
Flow chart for paper selection process.

Originally, 1152 records were found in the initial search through the query terms. After screening titles and abstracts for relevance, 75 papers were assessed for eligibility. Of these, 58 studies met the inclusion criteria after full-text evaluation and were included in the review. Figure 1 shows the flow chart of the paper selection process. In Supplementary Materials Table S1, we list the 58 selected studies, highlighting their sample sizes, the diseases addressed, and key findings.

The included studies had different designs, including prospective, retrospective, and observational approaches. A total of 47 studies focused on OSCC, 4 explored the role of TILs in OPMDs, and 7 compared TILs’ roles in both OSCC and OPMDs. Notably, most of the studies were published in recent years, indicating the increasing interest and relevance of this field.

## 4. Discussion

TILs have been identified as potentially valuable predictors of prognosis in OSCC patients. Some studies have suggested a correlation between TIL infiltration and clinical features.

TILs are primarily localized in the stroma rather than within the tumor itself. Generally, a low concentration of TILs has been significantly associated with a higher depth of invasion (DOI), the presence of lymph node metastasis, larger tumor size, and a higher frequency of lymphovascular invasion [16,17,18,19].

However, TILs comprise various types of immune cells with different characteristics, activities, and effects on tumors.

It is known that some TILs, such as T and B cells, can recognize tumor antigens (called tumor-associated antigens and tumor-specific antigens), triggering a tumor-specific adaptive immune response [20]. Others, like natural killer (NK) cells, react without prior sensitization by releasing cytokines through which they kill altered cells, including tumor cells. Additionally, NK cells help shape T and B cell adaptive immunity [21]. Notwithstanding the host immune system’s efforts—both innate and adaptive—it is often insufficient in controlling tumor growth, and the TME can be shaped to protect the tumor from immune reactions.

The TIL population includes T cells, B cells, and NK cells, which are further subdivided into different subgroups. The most studied subgroups can be summarized as follows: T cells are divided into CD8+ cells and CD4+ cells (including helper T cells and regulatory T cells—CD4+ CD25+ Foxp3+). B cells (CD45+ and CD19+) are divided into antigen-experienced B cells (CD27+), plasmablasts (CD38+), plasma cells (CD138+), and regulatory B cells—Bregs. Each of these TIL subgroups exhibits different functions.

### 4.1. T Lymphocytes

T cells, of which CD3 is the pan marker, play a central role in the adaptive immune response. T lymphocytes are also involved in tumor immunity and represent one of the main cell types in the immune infiltrate of the TME.

T lymphocytes are further divided into several subtypes, each with distinct activities and implications for tumor behavior. Moreover, the balance between different cell types (such as the CD4/CD8 ratio) is important, and valuable insights can be drawn from its analysis [22].

One of the main T cell subtypes are CD8+ T cells. The literature appears overall consistent recognizing the cytotoxic activity of CD8+ T cells in the TME of OSCC, as well as their consequently effective antitumor immunity [14,21,23,24].

A high concentration of CD8+ cells, both within the cancer nests (intratumoral) and in the stroma near the cancer invasion front (peritumoral), has been associated with better clinical outcomes in OSCC patients. Specifically, a higher density of peritumoral CD8+ cells is associated with a tendency toward longer survival times [25,26,27]. It has been observed that the higher the number of CD8+ cells, the lower the tumor mitotic index. The favorable relationship between CD8+ cells and prognosis (prolonged survival time) is consistent with findings in several other tumor types, including colorectal, gastric, esophageal, and head and neck cancers. Additionally, CD8+ concentration in the OSCC TME is elevated compared to normal mucosa [20,25,28,29,30,31,32]. When comparing different sites of OSCC, lip carcinoma shows a lower rate of local invasion and metastasis, which positively influences prognosis. Interestingly, in lip carcinoma, CD8+ cell concentration is significantly higher than in other oral cancer sites [25]. However, we have to report also some contradictory results regarding CD8+ concentration and metastasis. In some studies, no significant difference is found between metastatic and non-metastatic SCC [25,33], while in others, CD8+ cells have been associated with the absence of lymph node metastasis [28,34]. On the other hand, at metastatic sites, when present, the concentration of cytotoxic T cells is higher than at the primary tumor site [24].

Despite a few studies in contradiction, CD8+ expression has also been indicated as an independent prognostic factor in OSCC patients [28].

Not only the concentration but also the activation status of CD8+ T cells must be considered for a comprehensive evaluation. CD8+ T cells are abundant both in the stroma and in the tumor nests, but their activation status differs. CD8+ T cells in the tumor nests are mostly functionally suppressed, characterized by high expression of PD-1, an inhibitory receptor. In contrast, stromal CD8+ T cells are phenotypically activated, with high expression of NKG2D and Ki-67, an activating receptor and a proliferation-associated marker, respectively. The increased proliferative activity of stromal CD8+ T cells is likely related to the high number of mature dendritic cells (DCs) in the same microanatomic site, suggesting an active and ongoing immune response. An interesting conclusion is that within a single tumor, TILs exhibit different activation statuses depending on their microanatomic location [23]. A negative correlation between PD-L1 immunoexpression and the number of CD8+ T cells in the OSCC TME (associated with both better and poorer prognosis) has been reported. PD-L1 expression alone does not necessarily lead to a non-inflamed TME, but it may influence the efficacy of the immune response. This observation is consistent with recent evidence identifying the PD1:PD-L1 pathway as one of the major mechanisms for regulating tumor immunity, as it down-regulates cytotoxic T lymphocytes [27,29,35].

Another subgroup of T cells is represented by tissue-resident memory T cells (Trm), distinguished by double positivity for CD103 and CD8. Trm cells have been recognized for their pivotal role in antitumor immune responses in several cancer types, including OSCC. A high stromal concentration of Trm cells is associated with better prognosis and could serve as an independent prognostic indicator [36].

Along with CD8+, CD4+ T cells represent a major subtype of T cells. CD4+ T cells can be further subdivided into T helper cells (Th cells), CD4+ central memory T cells, and regulatory T cells (Tregs), each with distinct roles in the TME.

When looking at CD4+ cells, they behave in the opposite way to CD8+. The mean number of CD4+ T cells is significantly higher in OSCC cases with poorer prognosis compared to those with a better one [29,30]. Furthermore, A positive correlation between the immunoexpression of PD-L1 and the number of CD4+ T cells in OSCC with poor prognosis has been reported. Cancer cells may evade immune surveillance by expressing PD-L1, which induces apoptosis in PD-1-expressing T lymphocytes, particularly cytotoxic T cells. Tumors with higher PD-L1 expression also exhibit increased infiltration of CD4+ T cells, including immunosuppressive CD4+ Tregs, as well as higher levels of several cytokines, including CCL19, CCL21, CXCL9, CXCL10, CXCL13, and LTb [35,37,38].

Then, the concentration of CD4+ T cells can be influenced by individual behaviors. Specifically, regular alcohol consumption is associated with lower CD4+ T cell concentrations and poorer tumor differentiation [7].

However, CD4+ density is not significantly associated with clinicopathological features or prognosis [28,33].

Looking at the subtypes of CD4+ cells, Th cells and CD4+ central memory T cells typically contribute to tumor-specific adaptive immunity. Th cells, in turn, differentiate into several distinct subtypes, including Th1, Th2, Th17, and others. Each subtype secretes a specific cytokine profile and exerts different activities. Th1 cells are associated with a proinflammatory cytokine profile that is more prevalent in the early stages of cancer, while Th2 cells have an anti-inflammatory cytokine profile that increases in late-stage cancers. Consequently, different Th subtypes in the TME of OSCC can influence various tumor behaviors. Additionally, an imbalance in Th lineages has been reported in the OSCC TME compared to healthy individuals. Th1 cells and CD4+ central memory T cells have been associated with good prognosis, whereas anti-inflammatory Th2 cells are linked to poor prognosis [4,14,39,40]. Th17 cells (CD4+IL17A+) deserve separate consideration due to their high plasticity, which depends on the other cells present in the TME and leads to conflicting activities. Th17 cells can promote an antitumor response by facilitating the recruitment of other effector immune cells, but they can also enhance tumor cell growth, proliferation, and metastasis. As a result, in ovarian and prostate cancers, IL-17-positive cells (including Th17 cells and others) are positively correlated with survival, whereas this correlation is not observed in head and neck cancers. Specifically in OSCC, Th17 cell concentrations vary by site; they are typically more concentrated in lip SCC compared to tongue SCC, which may contribute to their differing levels of aggressiveness. Notably, Th17 cells retain the ability to differentiate into Tregs or Th1 cells, with opposite implications for tumor behavior [20,26,39,41].

Tregs are another CD4+ cell subtype. Tregs are characterized by the CD4+CD25+Foxp3+ phenotype. Foxp3 (forkhead box P3) is the most specific marker distinguishing Tregs from other T cells. Historically, the role of Tregs has been controversial, with studies yielding contradictory results; sometimes they were associated with worse prognosis, while other times with better locoregional control. The recent literature increasingly suggests that Tregs primarily have a protumoral role, although further studies are needed to fully elucidate their mechanisms of action.

The suppression of antitumor activity by CD4+ Tregs is thought to occur through a dual mechanism. On one hand, CD4+ Tregs inhibit the activation, proliferation, and effector functions of various cell types, including NK cells, CD4+ T cells, CD8+ T cells, B lymphocytes, and DCs, thereby contributing to tumor metastasis and progression in different cancer types. On the other hand, they secrete immunosuppressive cytokines that further suppress the activity of several immune cells, including NK cells, T cells, B cells, and DCs [9,13,16,24,32,42,43].

Furthermore, the immunoexpression of PD-L1 and the number of Foxp3+ Tregs appear to be proportional and higher in OSCC with poor prognosis compared to OSCC with better prognosis (and normal mucosa). Overexpression of PD-L1 has been observed in various solid tumors, including melanoma, colorectal cancer, lung cancer, pancreatic carcinoma, and hepatocellular carcinoma. The PD1:PD-L1 pathway is thought to play a central role in antigen-specific T cell response mediating PD-1-dependent immune suppression. Consequently, the data support the hypothesis that Tregs and PD-L1 are involved in OSCC development and progression [16,29,30].

The relationship between Treg concentration, tumor features, and clinicopathological endpoints has been analyzed. The data suggest a higher concentration of Tregs in smaller tumors (T1) and early stages of OSCC (when neoplastic cells are more immunogenic), allowing tumor cells to escape the antitumor immune response, particularly in older individuals [7,16,42,44,45]. A statistically higher number of Tregs has been found in low-grade tumors compared to high-grade ones [44]. However, within the intraepithelial region, Tregs appear more abundant in advanced clinical stages and in moderately/poorly differentiated tumors [10]. Tregs have also been associated with invasive behavior and depth of invasion [45]. A higher number of Tregs is reported in tumors with an intense inflammatory infiltrate. In contrast, a lower number of Tregs is observed in tumors arranged in small cell clusters [42,44,45]. A high density of Tregs also shows a negative impact on disease recurrence and disease-free survival (DFS) [8,43,46]. Furthermore, Tregs are more frequent in SCC samples than in adjacent non-neoplastic sites and in metastatic lymph nodes than in normal ones [47].

Tregs also appear to be related to patient age. Although Foxp3+ lymphocytes can be observed at all ages, Foxp3+ levels clearly tend to be higher in older patients, particularly older males. Notably, age has been identified as a potentially important confounding factor, which is not always fully considered and may partly explain the contradictory results of previous studies, where Tregs were sometimes associated with favorable prognosis and other times with unfavorable outcomes. This aspect requires further investigation [42].

In light of the evidence, Tregs expression in the OSCC TME has been proposed as an independent prognostic factor [47]. Nevertheless, as mentioned earlier, the role of Tregs remains complex and requires further elucidation. A recent study, in contrast, found that in tumors with a worse prognosis, Tregs tend to decrease, along with other cell types. However, the overall crosstalk between cells and the composition of the immune microenvironment must be taken into account. Indeed, Tregs can contribute to immune modulation, influencing tumor behavior [31].

A proportional expression of Tregs and Bregs has been observed, with the concentration of Bregs and Tregs inversely associated with the overall survival (OS) of OSCC patients [47].

Eventually, the last T lymphocytes subtype discussed is represented by a small subset called natural killer T (NKT) cells. The name ’NKT’ highlights that these cells express not only the classic T lymphocyte marker, the T cell receptor (TCR), but also the typical NK cell marker, CD56. NKT cells are involved in immune responses in various conditions, including autoimmunity, infections, and malignancies. Notably, NKT cells have been associated with antitumor activity. They have been evaluated in different malignancies, such as melanoma, head and neck cancer, colon, breast, and renal cancer, with a generally reduced number of NKT cells reported. In OSCC, there is a trend toward a decreased number of NKT cells in cases with poor prognosis compared to those with better prognosis [21].

### 4.2. B Lymphocytes

B cells, of which CD20 is the pan marker, produce antibodies as part of the humoral component of the adaptive immune system [14]. B cells also play a role in tumor immunity, although they are not very numerous in the TME and their exact relevance remains to be fully elucidated. Indeed, on one hand, in some studies, CD20+ B cells in OSCC TME have been identified as a significant prognostic factor for longer 5-year survival, and antigen-experienced B cells (CD27+) exert antitumor activity [10,20,35,48,49,50,51]. On the other hand, other studies have shown that the concentration of CD20+ cells increases with dysplasia grade, and intraepithelial infiltration at the invasive tumor front is directly associated with advanced tumor stage [30,52].

Of note, CD20+ cells are more abundant in patients who do not consume alcohol [20].

Mature B cells are divided into several subsets based on their location, cell surface phenotype, antigen specificity, cytokine secretion, and activation routes. One of the main B cell subtypes involved in tumor immunity is regulatory B cells (Bregs). Multiple markers have been proposed to identify Bregs, including CD1dhigh, CD5, CD19, CD24high, CD27 variable, CD38 variable, CD138 variable, IgMhigh, and IL-10. Although there is no consensus on a single marker or set of markers, one of the most commonly used sets is CD19+IL-10+. Under physiological conditions, Bregs help restrict excessive inflammatory responses. IL-10 is crucial for Bregs’ function as they inhibit pro-inflammatory cytokines (e.g., IFN-γ, IL-17), reduce the expression of MHC class II molecules, and support the differentiation of Tregs. Consequently, in the TME, Bregs are associated with a protumoral action, contrasting with the role of CD20+ [22,48]. The density of Bregs increases significantly compared to adjacent normal tissue, and their number is greater in metastatic nodes. They are associated with several clinicopathological features, including clinical stage and local and regional recurrence; however, they have not been correlated with age, gender, or T and N classification.

Furthermore, as mentioned before, the relationship between Bregs and Tregs has gained clinical relevance in recent studies and has been demonstrated in several tumor models. The concentration and distribution of Bregs appear related to Tregs. Overall, Bregs expression is a negative predictive factor, though it is not an independent prognostic factor but rather partially dependent on Tregs [50]. The proposed mechanism is that persistent inflammation at the tumor site can lead to local immunosuppression, thereby favoring tumor development [47].

### 4.3. Natural Killer

Natural killer (NK) cells are part of the innate immune system. Their cytotoxic activity does not require prior sensitization to antigens and is regulated by both germline-encoded receptors and the cytokine microenvironment. Cytokines can influence NK cells directly or by modulating other immune cells in the environment. NK cells also interact with DCs and play a role in antigen presentation, which modulates and shapes T cell responses.

In the TME, NK cells, due to their cytotoxic activity, are associated with effective antitumor immunity. A high intratumoral and peritumoral concentration of NK cells has been significantly positively associated with better prognosis and prolonged survival in OSCC (such as in various cancer types, including colorectal, gastric, and esophageal) [20,21,23,25,28].

NK cells are typically identified by the marker CD56+, while CD57+ is associated with highly mature cytotoxic NK cells.

The concentration and activation status of NK cells vary according to the microenvironmental site. Higher numbers of NK cells are generally present in the peritumoral region compared to the intratumoral region. Interestingly, NK cells are significantly more concentrated in lip SCC than in metastatic OSCC (though not significantly different from non-metastatic OSCC), and NK cells in non-metastatic OSCC tend to be higher than in metastatic ones. Additionally, resident NK cells in the tumor nests are phenotypically inactivated, whereas those in the stroma are activated. Furthermore, in OSCC patients, the peripheral blood NK cell count is lower compared to healthy controls [23,25,53].

A high concentration of CD57+ cells in the OSCC TME is associated with better prognosis. Specifically, high CD57+ concentration correlates with early clinical stages and the absence of lymph node metastasis. The different concentrations of NK cells in the TME may indicate varying responses to neoplastic cells. In fact, CD57+ cells have also been suggested as an independent prognostic factor [25,28,49].

### 4.4. TME Composition and Distribution of the Immune Cells

Over the years, various studies have investigated the overall composition of the immune infiltrate in the TME of OSCC, as well as its clinicopathological implications.

While the importance of considering the immune infiltrate in the TME and the relationship among its components is well-recognized, a comprehensive understanding of each cell type and their interactions remains to be fully elucidated.

Firstly, the available data suggest an antigen-driven immune response against tumor cells, which, however, leads to immune dysfunction. For instance, the TME exhibits an increase in both Th17 and Treg cells, despite their opposing activities, demonstrating that the TME itself supports immune cell presence and ultimately results in immune dysfunction in OSCC patients [20].

Significant correlations between different cell types in the TME have been reported. Levels of CD8+ and CD68+ cells (which is the macrophages pan marker) are directly and significantly associated, while Tregs are positively associated with the total number of CD4+ cells. Ratios between different cell types, such as the neutrophil–lymphocyte ratio (NLR), platelet–lymphocyte ratio (PLR), and lymphocyte–monocyte Ratio (LMR), have been considered. Since monocytes and lymphocytes have antitumoral effects, evaluating these ratios can provide useful information for patient evaluation and risk assessment. For instance, high NLR and PLR are associated with poor prognosis. However, these variables have not yet been applied as clinical decision tools and require further investigation [7,14].

Regarding the distribution of TILs, most are found around the tumor islands (peritumoral), with only a few infiltrating the tumor islands (intratumoral). T cells are significantly more numerous than B cells [20,26].

The concentration of cells in the TME has been compared with peripheral blood mononuclear cells (PBMCs). An increased number of CD8+ T cells and a decreased number of CD4+ T cells have been observed in TILs compared to PBMCs, resulting in a reversed CD4+/CD8+ ratio in the TME. Evaluating the functional state of T cells shows increased expression of PD-1+ and Tim-3+ in both CD4+ and CD8+ T cells compared with PBMCs, indicating an enrichment of exhausted T cells in tumors. Among CD4+ T cells, both Th17 and Tregs are significantly more abundant in tumors than in circulating peripheral blood. However, the ratios of Th17/Tregs do not show significant differences between tumors and PBMCs. B cells (CD45+ and CD19+), antigen-experienced B cells (CD27+), and Bregs (IL-10+) are significantly enriched in tumors compared to corresponding PBMCs. Interestingly, T cells moving from peripheral blood to the tumor shift from naive to effector/memory states, indicating an immune response driven by local neoantigens in the TME. However, the immune response seems ineffective, as TILs in tumor tissues show an exhausted phenotype (PD-1+, Tim-3+) in tumor tissues [14,20].

Finally, clinicopathological endpoints have been related to immune cell composition.

A high concentration of stromal TILs correlates positively and significantly with OS, disease-specific survival (DSS), and DFS. Conversely, a low percentage of stromal TILs (≤20%) at the invasive front is associated with significantly poorer survival. Intratumoral TILs are generally reported as having no promising prognostic value (for OS, DSS, or DFS), although some studies associate them with increased OS. High density of CD3+ T cells, both in the cancer nests and in the stroma, is associated with early T1–T2 stages (and vice versa, low density with advanced T3–T4 stages) and with the N0 stage. Notably, TIL evaluation (concentration and morphological distribution) can be easily conducted on routine HE slides and may be applicable in everyday analysis if confirmed to have relevant implications for treatment decisions.

Overall, T cell count is decreased in patients with recurrence or who die. The CD4/CD8 ratio, combined with nodal extracapsular spread, is associated with tumor recurrence but does not influence overall survival. Even so, it has been proposed as an independent prognostic factor. Additionally, also the Th17/Treg ratio appears to be a good independent prognostic factor for OSCC [7,9,20,54,55].

Highlighting the importance of TME composition, recent classifications of OSCC have been proposed based on the immune profile. OSCCs are divided into “non-T cell-inflamed cancers” (low-density inflammatory infiltrate, decreased HLA expression, and low-level IFN-γ expression in TILs) and “T cell-inflamed cancers” (with the opposite characteristics). Non-T-cell-inflamed cancers display an unfavorable prognosis regardless of confounders such as age, gender, grade, and tumor size [9]. Another classification divides OSCC into “immune-inflamed phenotypes” (presence of a dense immune cell infiltrate next to the tumor cells), “immune-excluded phenotypes” (abundant immune cells that do not penetrate the parenchyma and are retained in the stroma), and “immune-desert phenotypes” (almost total absence of immune cells in the parenchyma or stroma of the tumor) [26]. These classifications are frequently used in tumor evaluation, although a universally accepted classification is still lacking [5].

Although the data widely demonstrate the importance of the immune infiltrate in the TME for tumor development, there is still no unified interpretation or clear parameters for their routine use in OSCC treatment strategies. Currently, traditional parameters, including TNM classification and differentiation, remain the gold standard in clinical decision-making.

### 4.5. Mediators

As is well known, cytokines interact with surrounding cells, including those in the TME, influencing their behavior (from recruitment and activation status to their overall activity). The way cytokines affect cells might help explain some of the conflicting results seen in studies about the role of different cell types. Therefore, it is important to further understand the relationship between cells and cytokines [53].

Among the main cytokines involved in tumor immunity are IFN-γ, TGF-β, TNF-α, IL-10, and IL-17. Below, we provide a brief overview of their roles.

IFN-γ acts through both direct action on target cells and activation of the host immune system. In the TME, IFN-γ plays a key role in activating cellular immunity and stimulating an antitumor immune response. Its actions include cytostatic, pro-apoptotic, and antiproliferative effects. IFN-γ inhibits angiogenesis in tumor tissue, induces apoptosis in regulatory T cells, reduces cellular proliferation, and enhances the motility and killing capacity of CD8+ T cells [53,56]. Low levels of IFN-γ are associated with a protumoral cell profile, such as a high number of Tregs, and poor prognosis [9,13].

TGF-β regulates various cellular processes, including proliferation, differentiation, apoptosis, plasticity, and migration. In tumor immunity, it is associated with tumor growth through the upregulation of Tregs, which have a protumoral activity [13,57,58,59].

TNF-α is both an adipokine and a cytokine. As an adipokine, it is involved in insulin metabolism, while as a cytokine, in the inflammatory responses, it influences cell survival, proliferation, differentiation, and death. In cancer and carcinogenesis, dysregulation of TNF-α has been observed. TNF-α is secreted by multiple inflammatory cells and may have several sources in the TME. Its role in cancer is controversial. While most studies report that TNF-α promotes tumorigenesis by stimulating growth, proliferation, invasion, metastasis, and angiogenesis, some experimental systems suggest an antitumor effect, noting its ability to induce cancer cell death. Since TNF-α is involved in all stages of carcinogenesis, including cellular transformation, it has been suggested as a marker (along with IL-12β) for monitoring malignant transformation in some oral potentially malignant disorders (OPMDs), such as oral leukoplakia [53,60,61].

IL-10 displays both anti-inflammatory and immunostimulatory functions. In cancer, it may exert both pro- and antitumor effects depending on the overall context of the TME, including different immune cells, regulatory signals, and concentrations of multiple factors. The anticancer effect of IL-10 is attributed to its ability to support and stimulate CD8+ T cells and promote inflammation through the induction of pro-inflammatory cytokines (such as IFN-γ and granzyme B). Conversely, IL-10 could also negatively regulate pro-inflammatory cytokines like IL-6 and IL-12/IL-23, maintain the homeostasis of anti-inflammatory Tregs, and suppress pro-inflammatory Th17 cells, which contribute to tumor growth [61,62]. Higher levels of IL-10 have been reported in OSCC patients compared to healthy individuals. Additionally, high levels of IL-10 in peripheral blood have been associated with T3 and T4 stages and lymphatic metastasis, and IL-10 has been linked to poorer prognosis [13,53].

IL-17 is a pro-inflammatory cytokine produced by Th17 lymphocytes and macrophages. It stimulates the production of cytokines (IL-6, TGF-β, TNF-α), chemokines (such as IL-8 and GRO-α), and prostaglandins from various cells (fibroblasts, endothelial cells, keratinocytes). In the cancer TME, IL-17 exerts both pro- and antitumor effects, depending on the cellular source and the overall TME composition. While Th17 cells are associated with antitumor effects and are more concentrated in early-stage tumors (T1/T2), IL-17+ macrophages are linked to higher malignancy in OSCC. These findings suggest that a single cytokine, such as IL-17, can have different roles depending on the tumor context and the specific cell types that secrete it [20,41].

### 4.6. Infiltrating Lymphocytes in OPMDs

When considering carcinogenesis, there is considerable literature suggesting that inflammation plays a critical role in the progression from OPMDs to OSCC [63,64]. Among inflammatory components, some studies have evaluated the role of infiltrating lymphocytes (ILs) in OPMDs, both overall and by subtype, since these conditions often lead to OSCCs. ILs have been explored in OL, OLP, and AC. Additionally, ILs have been studied also in OED. These findings have been compared with TILs in OSCC.

Notably, ILs are almost always absent or only mildly present in non-dysplastic lesions, with their concentration gradually increasing in OED and reaching a maximum in OSCC. A similar trend is observed for PD-1 or PD-L1 positive cells, suggesting that the PD-1/PD-L1 pathway, along with IL involvement, may promote carcinogenesis early on. Consequently, they have been proposed as prognostic factors for the transformation of OPMDs [4,5,65].

Specifically, the main IL subtypes explored in OPMDs and OED include T cells (both CD8+ and CD4+ cells) and NK cells. Below, we summarize the main findings.

#### 4.6.1. T Lymphocytes

Among the OPMDs, CD8+ cells have been investigated mainly in OLP and OL with dysplasia (mild to moderate).

Generally speaking, OLP is characterized by a band-like inflammatory infiltrate of CD4+ and CD8+ lymphocytes in the submucosa, disruption of the basement membrane, and degeneration of the basal keratinocytes. This can be described as “an active immune-cell attack.” Interesting observations can be drawn from comparing the immune infiltrate behavior between OLP and OSCC. The number of CD8+ T cells in OLP is similar to that in cancer, both in the cancer nests versus OLP mucosa and in the cancer stroma versus OLP submucosa. However, their activation status differs. CD8+ T cells in the tumor nest are mostly functionally suppressed (with high expression of PD-1), whereas stromal cells and OLP T cells are phenotypically activated (with high expression of NKG2D and ki-67). The increased proliferative activity of CD8+ T cells in OLP is likely related to the high number of mature DCs, suggesting an active and ongoing immune response [23,46].

Different evidence has been found regarding OL with dysplasia. In these lesions, the concentration of CD8+ cells is lower than in OSCC, both in the peri- and intratumoral region, and is similar to that in healthy controls. One possible explanation is that the low number of CD8+ cells in OL could be favorable for the carcinogenesis process [25]. On the other hand, more recent evidence shows a positive correlation between CD8+ cells and dysplastic progression [46,66].

Furthermore, CD4+ cells, especially Tregs, have been evaluated in OPMD MEs and compared with the OSCC TME. The studies have included OLP, OL, OED (both low-grade (LD) and high-grade (HD) epithelial dysplasia), and AC.

The concentration of Tregs in OPMDs is positively associated with postoperative recurrence and progression from OPMD to OSCC. The proposed mechanism is that immunosuppressive Tregs down-regulate the induction of autoreactive T cells and maintain tolerance to self-antigens, thereby inhibiting autoimmune reactions. A positive gradient of Treg density is reported from OLP/OL to LD to HD to OSCC. These data suggest that infiltrating Tregs play a role in regulating epithelial cell behavior [45,46,66,67].

An interesting association between sex and inflammatory infiltration in OPMDs (Tregs and macrophages) has been reported, with higher levels observed in females. It has been suggested that this may be related to the higher prevalence of autoimmune diseases in females, which could be linked to sex-related cancerization and cancer progression. Overall, the data suggest that Tregs may promote aggressive behavior in both OSCC and OPMDs [45].

Regarding AC, the accumulation of functional Treg cells (CD4+ CD25+ FoxP3+) in the AC microenvironment has been demonstrated. One proposed mechanism is that the accumulation of Tregs may inhibit T cell proliferation (especially helper T cells) and favor high levels of immunosuppressive cytokines. Consequently, this microenvironment may contribute to recurrence, progression, and malignant transformation [68].

#### 4.6.2. Natural Killer

NK cells have been evaluated in OLP, with a focus on their concentration and activation status, and compared to those in OSCC. In OSCC, NK cells are abundant in both the cancer nests and the cancer stroma. In contrast, in OLP, the concentration of NK cells in the submucosa is similar to that in the cancer stroma, but they are nearly absent in the OLP mucosa. Regarding their activation status, NK cells in OLP are phenotypically activated, much like those in the tumor stroma, whereas NK cells within the tumor nest are phenotypically inactivated [23].

In leukoplakias with mild to moderate dysplasia, a lower number of NK cells has been found compared to OSCC. The NK cell count in dysplastic samples is similar to that in healthy tissue. It has been suggested that the low number of NK cells in OED lesions might contribute to the carcinogenesis process [25].

### 4.7. Infiltrating Lymphocytes Recap

The following tables summarize the findings discussed earlier, organized by cell type. Table 2 outlines TIL activities in OSCC. Table 3 presents IL activities in OPMDs.

### 4.8. Limitations

One limitation of this scoping review is that the increasing attention given to this area of research, combined with the rapid publication of new studies, may have led to the omission of some relevant works. Additionally, the review was based solely on findings from the PubMed database. Even if this approach may limit the comprehensiveness of the findings, we considered it appropriate for a scoping review.

## 5. Conclusions

This review emphasizes the crucial role of TILs in the ME of OSCC and OPMDs. Highlighting how the complex interactions between TME components and cancer cells are essential for tumor progression and prognosis. However, the complexity of the topic, coupled with the absence of universally accepted research standards, sometimes leads to seemingly contradictory findings.

In this context, it is worth noting that the concept of cancer as “a multidimensional spatiotemporal ecological and evolutionary unity” has been proposed in the literature, defining it as a “pathological ecosystem, a disease with ecological and evolutionary unity”. Within such a framework, it is “essential to apply ecological principles and approaches to study the etiology, pathogenesis, pathological changes, and outcomes”. The cells within this tumor ecosystem interact dynamically, influencing one another across spatial and temporal dimensions. This perspective on cancer highlights the plasticity of both tumor and stromal cells, offering a potential explanation for the variability in TIL behaviors and immune cell activity reported in the literature. Indeed, the functions of TME components should not be viewed as static or uniform across space and time but rather must be contextualized within the “tumoral ecosystem” [69].

In summary, the current evidence suggests that TILs, including CD8+ T cells, CD4+ T cells, Tregs, and NK cells, display distinct behaviors based on their location and the disease stage. In OSCC, a higher level of CD8+ T cells and mature cytotoxic NK cells (CD57+) correlates with better prognosis and longer survival, suggesting a strong antitumor immune response. Conversely, Tregs are associated with poorer outcomes, likely due to their role in suppressing effective antitumor immunity.

Research on OPMDs, such as OL and OLP, is limited, but some insights can be drawn. For instance, OLP shows a similar density of CD8+ T cells to OSCC, but their activation status differs. Additionally, OL with OED generally exhibits a significantly lower number of NK cells compared to OSCC.

The varying roles of cytokines and immune cells underscore the complexity of the TME in different stages and types of oral lesions. For example, contradictory findings regarding cytokines like IL-10 and TNF-α emphasize the need for more targeted research.

In conclusion, while the crucial role of TILs in carcinogenesis and OSCC development is well established and broadly accepted, our current understanding of the TME remains insufficient for clinical decision making.

As widely discussed, TILs encompass a broad range of cell types, each contributing to different and often contrasting outcomes. Despite various hypotheses, the prognostic value of TILs and thier association with the clinicopathological features in both OSCC and OPMDs remain uncertain [26,55]. Further research is needed to fully elucidate the mechanisms of TILs in the OSCC TME and even more so in the ME of OPMDs.

In the authors’ opinion, gaining a deeper understanding of this topic and clarifying the complex dynamics of TILs and the TME could offer new perspectives on both OSCC treatment and OPMD management. TILs could potentially serve as objective diagnostic criteria, which are currently lacking, especially in OPMDs. To this end, expanding current knowledge about the role of TILs in the TME of OSCC and OPMDs is essential.

## Figures and Tables

**Table 1:** Inclusion and exclusion criteria.

	Inclusion Criteria	Exclusion Criteria
Study design	Original research and clinical observational studies.	Case reports, editorials, letters, and reviews
Population	Human in vivo research focusing on OSCC or OPMDs.	In vitro research, animal model research, and studies that include pharyngeal diseases where data cannot be separated from the oral cavity.
Language	Publications in English.	Publications not in English or not available in full text.
Relevance	Studies that directly address the role of TILs in the context of OSCC or OPMDs.	Studies that do not provide specific data on TILs or do not meet the research question’s criteria.

**Table 2:** Tumor-infiltrating lymphocyte activities in OSCC.

Lymphocyte Subtype	Primary Activities	Prognostic Implication
CD8+ T Cells	Cytotoxic activity, killing tumor cells. Phenotypically activated in the stroma, with an active immune response.	Positive. High concentration linked to better prognosis and outcomes (longer survival time).
CD8+ Tissue-Resident Memory T Cells (Trm)	Cytotoxic activity (targeting tumor cells)	Positive. High concentration linked to better prognosis.
CD4+ Th1 Cells	Tumor-specific adaptive immunity. Pro-inflammatory cytokine profile. Promotes CD8+ T-cell activity	Positive. High concentration linked to better prognosis.
CD4+ Central Memory Cells	Contribute to adaptive immunity	Positive. High concentration linked to better prognosis.
CD4+ Th2 Cells	Anti-inflammatory cytokine profile. Supports humoral immunity	Negative. High concentration linked to poorer prognosis.
CD4+ Th17 Cells	High plasticity; can enhance immune cell recruitment or tumor growth. Can differentiate into Tregs or Th1 Cells.	Depends on TME composition and tumor site. Effects can be both protumor and antitumor depending on context.
CD4+ Regulatory T (Tregs) Cells	Immunosuppressive, inhibits CD8+ T Cells and NK Cells.	Negative. High concentration linked to worse outcomes (decreased overall survival).
NKT Cells	Antitumor immune responses.	Positive. High concentration linked to better prognosis.
CD20+ B Cells	Antibody production as part of the adaptive humoral immune response.	Positive. High concentration linked to better prognosis and outcomes (longer survival time).
Regulatory B (Bregs) Cells	Immunosuppressive, supports Tregs and suppresses T-cells.	Negative. High concentration linked to poorer prognosis (decreased overall survival, advanced stages, higher recurrence).
NK Cells	Innate immune system, direct cytotoxicity against tumor cells.	Positive. High concentration linked to better prognosis (longer survival time).

**Table 3:** Infiltrating lymphocyte activities in OPMDs.

Lymphocyte Subtype	Primary Activities	Prognostic Implication
CD8+ T Cells	Cytotoxic activity, active immune response. Phenotypically activated in mucosa and submucosa.	Positive. High concentration may indicate immune surveillance and delayed transformation.
CD4+ Tregs Cells	Immunosuppressive. Down-regulate autoreactive T cells.	Negative. High concentration linked to postoperative recurrence and progression to OSCC.
NK Cells	Innate immune system, direct cytotoxicity. Phenotypically activated in mucosa and submucosa.	Positive. High concentration may indicate immune surveillance and delayed transformation.

## Data Availability

Not applicable.

## Supplementary Materials

The following supporting information can be downloaded at: https://www.mdpi.com/article/10.3390/jcm14020606/s1, Table S1: Summary of Selected Studies (in chronological and alphabetical order).
