# Supplementary material for: Investigating Tumor-Infiltrating Lymphocytes in the Microenvironment of Oral Squamous Cell Carcinoma (OSCC) and Oral Potentially Malignant Disorders (OPMDs): Can They Shift Our Perspective? A Scoping Review
Source: J Clin Med. 2025 Jan 18;14(2):606. doi: 10.3390/jcm14020606 (PMC11766165; doi:10.3390/jcm14020606)
Supplement: Supplementary file 1 [file jcm-14-00606-s001.zip › jcm-3370458-supplementary.pdf]

Journal of
Clinical Medicine

MDPI

Supplementary Material

Investigating Tumor-Infiltrating Lymphocytes in the Microenvironment of Oral Squamous Cell Carcinoma (OSCC) and Oral Potentially Malignant Disorders (OPMDs): Can They Shift Our Perspective? A Scoping Review

Samuele Sutera *, Olga Anna Furchì and Monica Pentenero

Oral Medicine and Oral Oncology Unit, Department of Oncology, University of Turin, 10043 Turin, Italy; olgafurchi@gmail.com (O.A.F.); monica.pentenero@unito.it (M.P.)
* Correspondence: samuele.sutera@unito.it; Tel.: +39-339-261-3551

Table S1. Summary of Selected Studies (in chronological and alphabetical order).

Study	Sample size*	Disease(s)**	Key Finding (related to scoping review)
Katou, F., et al. 2007 [1]	29	OPMDs<br>OSCC	CD8+ cells number in OLP and OSCC is similar.<br>NK cells in cancer nest are far outnumbered those in the OLP mucosa, but in cancer stroma and OLP submucosa are similar.
Gasparoto, T.H., et al. 2010 [2]	22	OSCC	Tregs are associated to a negative prognosis.
Zancope, E., et al. 2010 [3]	108	OPMDs<br>OSCC	CD8+ and NK are significantly higher in OSCC than OL, AC, and controls.
Dayan, D., et al. 2012 [4]	64	OSCC	Tregs (FoxP3+) have a negative impact on disease recurrence.
Gasparoto, T.H., et al. 2014 [5]	24	OPMDs	No significant difference between patients with AC and healthy subjects in the percentage of CD3+CD4+, CD3+CD8+, and CD4+CD25+ cells in PBMC.

			AC exhibited accumulation of the CD3+ T cells, CD4+ T cells, CD8+ T cells, CD19+ B cells, CD4 CD25 T cells, and CD8 CD25 T cells.
Dutta, A., et al. 2015 [6]	150	OSCC	In tumor tissue, NK cells are negatively regulated and appear to be in an inactivated state.
Wolf, G.T., et al. 2015 [7]	39	OSCC	TILs subset is not statistically correlated to overall prognosis in surgical treated patients.
da Cunha, F.A.F., et al. 2016 [8]	50	OSCC	Lower Lip SCC with an intense inflammatory infiltrate exhibit a larger median number of Tregs than tumor with moderate or absent inflammatory infiltrate; it could be related to cancer development in early stage.
Zhou, X., et al. 2016 [9]	46	OSCC	Both Bregs and Tregs number is significantly higher in OSCC areas and increase in metastatic nodes.
Caldeira, P.C., et al. 2017 [10]	26	OSCC	There is a higher percentage of circulating neutrophils, lower percentage of lymphocytes, and a higher NLR in patients with OSCC than healthy individuals.<br>Lower amount of circulating neutrophils, higher lymphocytes, and lower NLR are related with better outcomes (DFS).
Fang, J., et al. 2017 [11]	78	OSCC	High CD+ and CD57+ cells are significantly associated with longer survival.
Mattox, A.K., et al. 2017 [12]	53	OSCC	PD-L1 expression is positively correlated with moderate/high CD4+PD-1+ and CD8+PD-1+ TILs density, but it is not significantly associated with clinical outcomes.
Stasikowska-Kanicka, O., et al. 2017 [13]	78	OSCC	Higher CD56+ cells concentration is associated to better prognosis and OS.
Stasikowska-Kanicka, O., et al. 2018 [14]	96	OSCC	CD8+ cells are significantly more concentrated in OSCC with better prognosis.<br>FoxP3+ cells are significantly more concentrated in OSCC with poor prognosis.
Wirsing, A.M., et al. 2018 [15]	75	OSCC	Tumor with high pD-L1 expression show increased infiltration of CD4+ and CD8+ cells compared to those with low PD-L1 expression.<br>The high-endothelial venule-negative tumors are less infiltrated with CD+ and CD20+ cells, than positive ones.<br>High-endothelial venules and low T stage are independent positive prognostic factors.
Boxberg, M., et al. 2019 [16]	66	OSCC	High concentration of CD4+FoxP3+ cells is an independent prognostic factor for favorable OS, DSS, and DFS.<br>High CD8/CD4 ratio is an independent prognostic factor for favorable DSS, and DFS.
Heikkinen, I., et al. 2019 [17]	308	OSCC	Low percentage of stromal TILs (≤ 20%) at the invasive front have a significantly poor survival (OS, DSS, and DFS).
Kouketsu, A., et al. 2019 [18]	127	OPMDs<br>OSCC	There is a positive gradient of CD25+, FoxP3+, CD163+, and CD204+ cells correlating with the increasing malignancy of the lesion, from OPMDs to OSCC.
Xiao, Y., et al. 2019 [19]	44	OSCC	Higher stromal CD103+CD8+ TIL density, higher intratumoral CD103+DC cells, and higher stromal Trm density indicate a favorable prognosis.

Amaral, M.G.D., et al. 2020 [20]	48	OSCC	FoxP3+ are less concentrated in young individuals.
Dar, A.A., et al. 2020 [21]	75	OSCC	MDSCs suppress T-cell proliferation, thereby impairing antitumor immunity and promoting tumor progression in OSCC patients.
Mukherjee, G., et al. 2020 [22]	94	OSCC	High CD3+ and CD8+ cells concentration is associated with small tumor size, higher OS, and inversely related to lymph node metastasis.
Quan, H., et al. 2020 [23]	242	OSCC	High Th17/Tregs ratios is significantly associated to higher OS rates.<br>Bregs may contribute to immunosuppression in the TME.<br>CD4+ and CD8+ cells are not associated with any clinical factors<br>CD20+ cells level are positively associated with long OS.
Troiano, G., et al. 2020 [24]	211	OSCC	High level of CD8+ cells correlate with longer OS.<br>Immune-desert phenotype is correlated with lower DFS, DSS, and OS.
Bezerra, T.M.M., et al. 2021 [25]	68	OSCC	Th17 cells have a great plasticity, according to the TME, and show both pro and anti-tumor activity.
Chatzopoulos, K., et al. 2021 [26]	16	OSCC	TILs density is inversely related to lymphovascular invasion.<br>Tregs facilitate tumor immune escape, tumor growth, and an immunosuppressive TME.
Fraga, M., et al. 2021 [27]	31	OSCC	Immunomodulatory changes induced by the TME involve CCR8 expression and regulatory Th2 phenotypes.
Hori, Y., et al. 2021 [28]	62	OSCC	Higher density of FoxP3+ T cells and CD163+ macrophages are related with lower DFS.
Klein, M., et al. 2021 [29]	58	OSCC	No significant correlation has been found between the immunomarkers of tumor cells (PD-1), concentration of CD4, CD8, FOXP3, and lymph node metastasis or follow up outcome.<br>PD-L1 is higher expressed in patients with lymph node metastasis.
Xie, P., et al. 2021 [30]	79	OSCC	High concentration of Myeloid Dendritic cells, T cells CD4+ T central memory, and Myeloid progenitor are related with better prognosis.
Xu, B., et al. 2021 [31]	329	OSCC	Low stromal TILs concentration is associated to higher frequency of lymphovascular invasion.
de Souza, V.G., et al. 2022 [32]	33	OPMDs<br>OSCC	TILs show a positive gradient from benign lesion to OSCC.
Gaafar, N.M., et al. 2022 (a) [33]	45	OSCC	Tregs are associated with advance clinical tumor stage and moderately/poorly differentiated tumor.<br>B-cells have a potential positive role in OSCC early stages.
Gaafar, N.M., et al. 2022 (b) [34]	22	OSCC	CD4+ cells and PD-L1+ cells are associated with poor OS.<br>CD20+ intraepithelial infiltration at the ITF is associated with advanced tumor stage.<br>Increased CD8+ T cells are associated to low Tumor Budding.
Gan, C.P., et al. 2022 [35]	127	OPMDs	T and B cells increase in moderate-severe OED. CD8+ cells increase is not statistically significant.
Kujan, O., et al. 2022 [36]	101	OPMDs	There is a positive gradient of TILs concentration from non-dysplastic lesions to OSCC.

		OSCC	
Noda, Y., et al. 2022 [37]	186	OSCC	Low TILs concentration is significantly associated with DOI>10mm, advanced tumor stage, lymphovascular invasion, and lymph node metastasis.
Ruiz-Ranz, M., et al. 2022 [38]	348	OSCC	NLR, LMR, and SII may serve as valuable systemic markers to predict OSCC patient survival.<br>TILs concentration in TME and systemic inflammatory markers are significantly correlated.
Surendran, S., et al. 2022 [39]	270	OPMDs<br>OSCC	CD8+ cells is positively correlated with dysplastic progression.<br>A trend indicating that lower levels of Tregs recruitment are associated with improved survival (not statistically significant).
Wang, Z., et al. 2022 [40]	18	OSCC	High expression of CCDC43 inhibit immune-activated cells (B cells, CD8+ T cells, NK T cells, dendritic cells)
Ahuja, S., et al. 2023 [41]	51	OSCC	Low concentration of TILs is associated to T3/T4 and more frequent lymphovascular invasion.
Chang, S.R., et al. 2023 [42]	54	OSCC	Tumors with worse prognosis exhibited reduced memory B cells, CD8+ T cells, and regulatory T cells (Tregs), along with increased resting NK cells and M0 macrophages.
Daroonpan, P., et al. 2023 [43]	60	OSCC	Low CD8+/Treg ratio is associated with poor prognosis<br>Higher CD8+ concentration and CD8+/Tregs ratio is related to better OS.
Huang, Z., et al. 2023 [44]	80	OSCC	Higher infiltration of CD57+ and CD20+ is associated with better OS.<br>CD8+ concentration is not significant associated with OS and DFS.
Ikeuchi, Y., et al. 2023 [45]	69	OSCC	Higher concentration of CD8+ cells and FoxP3+ cells are related to significantly better DSS.
Soopanit, T., et al. 2023 [46]	64	OSCC	Low density of TILs is correlated with perineural invasion, while their high density with better DFS.
Sukhera, Z.A., et al. 2023 [47]	100	OSCC	Higher concentration of CD8+ is significantly associated to absence od lymph node metastasis and early clinical stage.
Thomas, A., et al. 2023 [48]	60	OPMDs<br>OSCC	CD20+ concentration shows a significant correlation with diagnosis (increasing in advanced stages of OSCC) and the subject's age group.
Wang, B., et al. 2023 [49]	80	OSCC	CD56+ are more concentrated in tumor nest than border and stroma regions.
William, W.N., Jr., et al. 2023 [50]	188	OPMDs	PD-L1 expression and CD3/8+ Lymphocyte have a strong correlation.
Wu, K., et al. 2023 [51]	11	OSCC	CD4+ T cells concentration descrease in Lymph Node+ than Lymph Node-.<br>Lymph Node metastasis and recurrende are associated with increases in PD-1 and glycolysis.
Yao, S., et al. 2023 [52]	400	OSCC	Infiltration abundance of memory CD4 T cells, resting NK cells, M0 macrophages, dendritic cells activated in tumor sample is significantly higher than normal samples.<br>High naïve B cell infiltration is associated with better prognosis.<br>High memory B cells infiltration is associated with poorer prognosis.

Yorozu, A., et al. 2023 [53]	47	OSCC	Higher CXCL12 expression tends to correlate with CD8+ T cells infiltration and better outcomes (though this relation is not statistically significant).
Bag, S., et al. 2024 [54]	2	OSCC	Tumor infiltrating CD20+ and CD8+ are associated with better prognosis.
Chang, Y.M., et al. 2024 [55]	162	OSCC	Category III (TSR≥50% and sTILs <20%) patients have poorest OS and DSS.
Flores-Hidalgo, A., et al. 2024 [56]	20	OPMDs	In OED, CD8+ cells infiltrate the dysplastic epithelium, correlating with dysplasia severity, while CD4+ lymphocytes show a mild increase in the basal layer.<br>Compared to OLP, there is a moderate increase in the inflammatory infiltrate in OED.
Rodrigues, R.R., et al. 2024 [57]	60	OSCC	T-bet+ cells are more concentrated in Tongue SCC than Lower Lip SCC but are not related to OSCC aggressiveness.
Steffen, C., et al 2024 [58]	9	OSCC	OSCC exhibits spatial intratumoral heterogeneity, highlighting the importance of analyzing multiple tumor regions.<br>Patients with recurrences show a notably increased expression of FoxP3, vimentin, IDO, CD4, CD68, and CD163 at the tumor margin.

*Sample size: The total number of participants is considered, including controls when available, as well as the sum of the diseases when multiple conditions are studied.

**Disease(s): Diseases are summarized as OSCC and/or OPMDs, without specifying when the considered study differentiates between various tumor sites or focuses on a specific site (e.g., Oral Tongue SCC, Lower Lip SCC), or when one or more specific OPMDs are considered (e.g., OL, OLP, AC). Additionally, in this context, OED is included within OPMDs. The last column provides further details when relevant to the present review objectives.

Abbreviations used in the table S1 are defined as follows: AC – Actin Cheilitis, DFS - Disease-Free Survival, DSS - Disease-Specific Survival, ITF – Invasive Tumor Front, LMR - lymphocyte-monocyte ratio, MDSCs - myeloid-derived suppressor cells, NLR - Neutrophil-lymphocyte ratio, OED – Oral Epithelial Dysplasya, OL – Oral Leukoplakia, OLP – Oral Lichen Planus, OPMDs – Oral Potentially Malignant Disorders, OS - Overall Survival, OSCC – Oral Squamous Cell Carcinoma, PBMC - peripheral blood mononuclear cells, SII - systemic immune-inflammation index, TME – Tumor Microenvironment, TSR - Tumor Stromal Ratio.

References

1. Katou, F., et al., *Differing phenotypes between intraepithelial and stromal lymphocytes in early-stage tongue cancer.* Cancer Res, 2007. **67**(23): p. 11195-201.
2. Gasparoto, T.H., et al., *Patients with oral squamous cell carcinoma are characterized by increased frequency of suppressive regulatory T cells in the blood and tumor microenvironment.* Cancer Immunol Immunother, 2010. **59**(6): p. 819-28.
3. Zancope, E., et al., *Differential infiltration of CD8+ and NK cells in lip and oral cavity squamous cell carcinoma.* J Oral Pathol Med, 2010. **39**(2): p. 162-7.
4. Dayan, D., et al., *Molecular crosstalk between cancer cells and tumor microenvironment components suggests potential targets for new therapeutic approaches in mobile tongue cancer.* Cancer Med, 2012. **1**(2): p. 128-40.
5. Gasparoto, T.H., et al., *Regulatory T cells in the actinic cheilitis.* J Oral Pathol Med, 2014. **43**(10): p. 754-60.
6. Dutta, A., et al., *Negative regulation of natural killer cell in tumor tissue and peripheral blood of oral squamous cell carcinoma.* Cytokine, 2015. **76**(2): p. 123-130.
7. Wolf, G.T., et al., *Tumor infiltrating lymphocytes (TIL) and prognosis in oral cavity squamous carcinoma: a preliminary study.* Oral Oncol, 2015. **51**(1): p. 90-5.
8. da Cunha, F.A.F., et al., *Immunohistochemical analysis of FoxP3+ regulatory T cells in lower lip squamous cell carcinomas.* Braz Oral Res, 2016. **30**(1): p. e130.
9. Zhou, X., et al., *CD19(+)IL-10(+) regulatory B cells affect survival of tongue squamous cell carcinoma patients and induce resting CD4(+) T cells to CD4(+)Foxp3(+) regulatory T cells.* Oral Oncol, 2016. **53**: p. 27-35.
10. Caldeira, P.C., et al., *Immunophenotype of neutrophils in oral squamous cell carcinoma patients.* J Oral Pathol Med, 2017. **46**(9): p. 703-709.
11. Fang, J., et al., *Prognostic significance of tumor infiltrating immune cells in oral squamous cell carcinoma.* BMC Cancer, 2017. **17**(1): p. 375.
12. Mattox, A.K., et al., *PD-1 Expression in Head and Neck Squamous Cell Carcinomas Derives Primarily from Functionally Anergic CD4(+) TILs in the Presence of PD-L1(+) TAMs.* Cancer Res, 2017. **77**(22): p. 6365-6374.
13. Stasikowska-Kanicka, O., M. Wągrowska-Danilewicz, and M. Danilewicz, *Association of infiltrating cells with microvessel density in oral squamous cell carcinoma.* Pol J Pathol, 2017. **68**(1): p. 40-48.
14. Stasikowska-Kanicka, O., M. Wągrowska-Danilewicz, and M. Danilewicz, *Immunohistochemical Analysis of Foxp3(+), CD4(+), CD8(+) Cell Infiltrates and PD-L1 in Oral Squamous Cell Carcinoma.* Pathol Oncol Res, 2018. **24**(3): p. 497-505.
15. Wirsing, A.M., et al., *Presence of high-endothelial venules correlates with a favorable immune microenvironment in oral squamous cell carcinoma.* Mod Pathol, 2018. **31**(6): p. 910-922.
16. Boxberg, M., et al., *Composition and Clinical Impact of the Immunologic Tumor Microenvironment in Oral Squamous Cell Carcinoma.* J Immunol, 2019. **202**(1): p. 278-291.
17. Heikkinen, I., et al., *Assessment of Tumor-infiltrating Lymphocytes Predicts the Behavior of Early-stage Oral Tongue Cancer.* Am J Surg Pathol, 2019. **43**(10): p. 1392-1396.
18. Kouketsu, A., et al., *Regulatory T cells and M2-polarized tumour-associated macrophages are associated with the oncogenesis and progression of oral squamous cell carcinoma.* Int J Oral Maxillofac Surg, 2019. **48**(10): p. 1279-1288.
19. Xiao, Y., et al., *CD103(+) T and Dendritic Cells Indicate a Favorable Prognosis in Oral Cancer.* J Dent Res, 2019. **98**(13): p. 1480-1487.
20. Amaral, M.G.D., et al., *FoxP3+ regulatory T cells in oral tongue squamous cell carcinoma in young and older patients.* Braz Oral Res, 2020. **34**: p. e096.
21. Dar, A.A., et al., *Myeloid-derived suppressor cells impede T cell functionality and promote Th17 differentiation in oral squamous cell carcinoma.* Cancer Immunol Immunother, 2020. **69**(6): p. 1071-1086.
22. Mukherjee, G., et al., *Density of CD3+ and CD8+ cells in gingivo-buccal oral squamous cell carcinoma is associated with lymph node metastases and survival.* PLoS One, 2020. **15**(11): p. e0242058.

23. Quan, H., et al., *The repertoire of tumor-infiltrating lymphocytes within the microenvironment of oral squamous cell carcinoma reveals immune dysfunction.* Cancer Immunol Immunother, 2020. **69**(3): p. 465-476.
24. Troiano, G., et al., *The immune phenotype of tongue squamous cell carcinoma predicts early relapse and poor prognosis.* Cancer Med, 2020. **9**(22): p. 8333-8344.
25. Bezerra, T.M.M., et al., *Assessment of the presence of interleukin 17(+) macrophages and Th17 cells in situ in lip and oral tongue cancer.* Hum Immunol, 2021. **82**(12): p. 945-949.
26. Chatzopoulos, K., et al., *Transcriptomic and Immunophenotypic Characterization of Tumor Immune Microenvironment in Squamous Cell Carcinoma of the Oral Tongue.* Head Neck Pathol, 2021. **15**(2): p. 509-522.
27. Fraga, M., et al., *Immunomodulation of T Helper Cells by Tumor Microenvironment in Oral Cancer Is Associated With CCR8 Expression and Rapid Membrane Vitamin D Signaling Pathway.* Front Immunol, 2021. **12**: p. 643298.
28. Hori, Y., et al., *Prognostic Role of Tumor-Infiltrating Lymphocytes and Tumor Budding in Early Oral Tongue Carcinoma.* Laryngoscope, 2021. **131**(11): p. 2512-2518.
29. Klein, M., et al., *Immune checkpoint analysis in lip cancer.* J Craniomaxillofac Surg, 2021. **49**(10): p. 950-958.
30. Xie, P., et al., *Identification of Candidate Target Genes and Immune Cells in Oral Squamous Cell Carcinoma.* Comput Math Methods Med, 2021. **2021**: p. 5802110.
31. Xu, B., et al., *Histologic evaluation of host immune microenvironment and its prognostic significance in oral tongue squamous cell carcinoma: a comparative study on lymphocytic host response (LHR) and tumor infiltrating lymphocytes (TILs).* Pathol Res Pract, 2021. **228**: p. 153473.
32. de Souza, V.G., et al., *Potential Histopathological and Immune Biomarkers in Malignant and Non-Malignant Oral Lesions.* J Oral Maxillofac Res, 2022. **13**(4): p. e3.
33. Gaafar, N.M., et al., *Characterization of immune cell infiltrate in tumor stroma and epithelial compartments in oral squamous cell carcinomas of Sudanese patients.* Clin Exp Dent Res, 2022. **8**(1): p. 130-140.
34. Gaafar, N.M., et al., *Epithelial PD-L1 expression at tumor front predicts overall survival in a cohort of oral squamous cell carcinomas from Sudan.* Clin Exp Dent Res, 2022. **8**(6): p. 1467-1477.
35. Gan, C.P., et al., *Transcriptional analysis highlights three distinct immune profiles of high-risk oral epithelial dysplasia.* Front Immunol, 2022. **13**: p. 954567.
36. Kujan, O., et al., *PD-1/PD-L1, Treg-related proteins, and tumour-infiltrating lymphocytes are associated with the development of oral squamous cell carcinoma.* Pathology, 2022. **54**(4): p. 409-416.
37. Noda, Y., et al., *Novel pathological predictive factors for extranodal extension in oral squamous cell carcinoma: a retrospective cohort study based on tumor budding, desmoplastic reaction, tumor-infiltrating lymphocytes, and depth of invasion.* BMC Cancer, 2022. **22**(1): p. 402.
38. Ruiz-Ranz, M., et al., *Prognostic implications of preoperative systemic inflammatory markers in oral squamous cell carcinoma, and correlations with the local immune tumor microenvironment.* Front Immunol, 2022. **13**: p. 941351.
39. Surendran, S., et al., *T-Cell Infiltration and Immune Checkpoint Expression Increase in Oral Cavity Premalignant and Malignant Disorders.* Biomedicines, 2022. **10**(8).
40. Wang, Z., et al., *Single-Cell Profiling Reveals Heterogeneity of Primary and Lymph Node Metastatic Tumors and Immune Cell Populations and Discovers Important Prognostic Significance of CCDC43 in Oral Squamous Cell Carcinoma.* Front Immunol, 2022. **13**: p. 843322.
41. Ahuja, S., et al., *Tumor-infiltrating lymphocytes in oral cavity squamous cell carcinoma and its association with clinicopathological parameters.* Pathol Res Pract, 2023. **251**: p. 154882.
42. Chang, S.R., et al., *The Concordant Disruption of B7/CD28 Immune Regulators Predicts the Prognosis of Oral Carcinomas.* Int J Mol Sci, 2023. **24**(6).
43. Daroonpan, P., et al., *Personal immune profiles: Diversity and prognostic value for oral tongue squamous cell carcinoma evaluated by comprehensive immune parameter analyses with multiplex immunofluorescence.* Oral Oncol, 2023. **143**: p. 106458.
44. Huang, Z., et al., *Prognostic value of tumor-infiltrating immune cells in clinical early-stage oral squamous cell carcinoma.* J Oral Pathol Med, 2023. **52**(5): p. 372-380.
45. Ikeuchi, Y., et al., *Immunohistological evaluation of patients treated with intra-arterial chemoradiotherapy and surgery for oral cancer.* Med Mol Morphol, 2023. **56**(4): p. 288-296.

46. Soopanit, T., et al., *Prognostic value and clinicopathological status of PD-L1 expression and CD8+ TILs in oral squamous cell cancer patients with or without traditional risk factors.* Head Neck, 2023. **45**(4): p. 1017-1025.
47. Sukhera, Z.A., et al., *Role Of Cd8+ Tumour-Infiltrating Lymphocytes In Predicting Regional Lymph Node Metastasis In Lip And Oral Cavity Squamous Cell Carcinoma.* J Ayub Med Coll Abbottabad, 2023. **35**(2): p. 288-293.
48. Thomas, A., et al., *Expression of CD 20 B-Lymphocyte in oral epithelial dysplasia and oral squamous cell carcinoma: A comparative immunohistochemistry study.* J Oral Maxillofac Pathol, 2023. **27**(2): p. 323-327.
49. Wang, B., et al., *Co-inhibition of adenosine 2b receptor and programmed death-ligand 1 promotes the recruitment and cytotoxicity of natural killer cells in oral squamous cell carcinoma.* PeerJ, 2023. **11**: p. e15922.
50. William, W.N., Jr., et al., *Spatial PD-L1, immune-cell microenvironment, and genomic copy-number alteration patterns and drivers of invasive-disease transition in prospective oral precancer cohort.* Cancer, 2023. **129**(5): p. 714-727.
51. Wu, K., et al., *Increased levels of PD1 and glycolysis in CD4(+) T cells are positively associated with lymph node metastasis in OSCC.* BMC Oral Health, 2023. **23**(1): p. 356.
52. Yao, S., et al., *CD79A work as a potential target for the prognosis of patients with OSCC: analysis of immune cell infiltration in oral squamous cell carcinoma based on the CIBERSORTx deconvolution algorithm.* BMC Oral Health, 2023. **23**(1): p. 411.
53. Yorozu, A., et al., *CXCL12 is expressed by skeletal muscle cells in tongue oral squamous cell carcinoma.* Cancer Med, 2023. **12**(5): p. 5953-5963.
54. Bag, S., et al., *Impact of spatial metabolomics on immune-microenvironment in oral cancer prognosis: a clinical report.* Mol Cell Biochem, 2024. **479**(1): p. 41-49.
55. Chang, Y.M. and C.C. Lee, *Stromal categorization of recurrent oral cancer after salvage surgery is associated with survival rates.* Eur J Surg Oncol, 2024. **50**(3): p. 108009.
56. Flores-Hidalgo, A., et al., *Immunophenotypic and Gene Expression Analyses of the Inflammatory Microenvironment in High-Grade Oral Epithelial Dysplasia and Oral Lichen Planus.* Head Neck Pathol, 2024. **18**(1): p. 17.
57. Rodrigues, R.R., et al., *Evaluation of the presence of Th1 response through T-bet and IFN-gamma immunohistochemical expression in lower lip and oral tongue squamous cell carcinomas.* Pathol Res Pract, 2024. **253**: p. 155010.
58. Steffen, C., et al., *Spatial heterogeneity of tumor cells and the tissue microenvironment in oral squamous cell carcinoma.* Oral Surg Oral Med Oral Pathol Oral Radiol, 2024. **137**(4): p. 379-390.
